# Identification of Epithelial-Mesenchymal Transition- (EMT-) Related LncRNA for Prognostic Prediction and Risk Stratification in Esophageal Squamous Cell Carcinoma

**DOI:** 10.1155/2021/5340240

**Published:** 2021-10-19

**Authors:** Peipei Wang, Yueyun Chen, Yue Zheng, Yang Fu, Zhenyu Ding

**Affiliations:** Department of Biotherapy, Cancer Center, West China Hospital, West China Medical School, State Key Laboratory of Biotherapy, Sichuan University, Chengdu, China

## Abstract

**Background:**

Epithelial-mesenchymal transition (EMT) is significantly associated with the invasion and development of esophageal squamous cell carcinoma (ESCC). However, the importance of EMT-related long noncoding RNA (lncRNA) is little known in ESCC.

**Methods:**

GSE53624 (*N* = 119) and GSE53622 (*N* = 60) datasets retrieved from the Gene Expression Omnibus (GEO) database were used as training and external validation cohorts, respectively. GSE53624 and GSE53622 datasets were all sampled from China. Then, the prognostic value of EMT-related lncRNA was comprehensively investigated by weighted coexpression network analysis (WGCNA) and COX regression model.

**Results:**

High expression of PLA2G4E-AS1, AC063976.1, and LINC01592 significantly correlated with the favorable overall survival (OS) of ESCC patients, and LINC01592 had the greatest contribution to OS. Importantly, ESCC patients were divided into low- and high-risk groups based on the optimal cut-off value of risk score estimated by the multivariate COX regression model of these three lncRNA. Patients with high risk had a shorter OS rate and restricted mean survival time (RMST) than those with low risk. Moreover, univariate and multivariate COX regression revealed that risk stratification, age, and TNM were independent prognostic predictors, which were used to construct a nomogram model for individualized and visualized prognosis prediction of ESCC patients. The calibration curves and time-dependent ROC curves in the training and validation cohorts suggested that the nomogram model had a good performance. Interestingly, clear trends indicated that risk score positively correlated with tumor microenvironment (TME) scores and immune checkpoints TIGIT, CTLA4, and BTLA. In addition, the Kyoto Encyclopedia of Genes and Genomes (KEGG) showed that PLA2G4E-AS1, AC063976.1, and LINC01592 were primarily associated with TNF signaling pathway, NF-kappa B signaling pathway, and ECM-receptor interaction.

**Conclusion:**

We developed EMT-related lncRNA PLA2G4E-AS1, AC063976.1, and LINC01592 for prognostic prediction and risk stratification of Chinese ESCC patients, which might provide deep insight for personalized prognosis prediction in Chinese ESCC patients and be potential biomarkers for designing novel therapy.

## 1. Introduction

Esophageal cancer is one of the top ten malignant tumors globally and the sixth leading cause of cancer-related deaths. Esophageal cancer is mainly prevalent in eastern Asia and eastern and southern Africa [1, 2]. Esophageal squamous cell carcinoma (ESCC) is the most common histological subtype of esophageal cancer, accounting for about 90%. ESCC has a high tendency to be aggressive and metastatic, as well as a high chance of recurrence. Even with multimodality treatments (surgery, radiotherapy, chemotherapy, and targeted therapy), the prognosis of ESCC patients remains poor [3]. Insight into the molecules and mechanisms behind ESCC invasion and metastasis helps deepen the understanding of the disease. It is also urgent to discover novel biomarkers to develop new therapeutic strategies and improve the prognosis of ESCC patients.

Long noncoding RNA (LncRNA) is a group of evolutionarily conserved RNA molecules with more than 200 nucleotides in length, lacking protein-coding ability [4]. Abnormal expression of LncRNA plays a vital role in the occurrence and development of various tumors [5–9], including ESCC [10, 11]. Many studies have shown that lncRNA plays multiple roles in malignant behaviors such as tumor formation, invasion, migration, and immunogenicity. LncRNA BRCAT54 inhibits the tumorigenesis of non-small -ell lung cancer by binding to RPS9 to regulate JAK-STAT and calcium pathways [12]. Moreover, lncRNA LINC00472 regulates cell stiffness and inhibits the migration and invasion of lung adenocarcinoma by binding to YBX1 [13]. In addition, a study found that LIMIT may be a target for cancer immunotherapy [14]. LncRNA can participate in the occurrence and development of tumors by affecting chromatin remodeling, histone modification, DNA methylation, gene transcription, translation, etc.

Notably, the effect of lncRNA on the epithelial-mesenchymal transition (EMT) of tumor cells has also been confirmed in recent studies [15, 16]. EMT is an essential step in the metastasis of malignant tumors, which can transform epithelial-like cells into a mesenchymal-like cell state. By modifying the adhesion molecules expressed in cells, EMT reduces the adhesion ability of epithelial-derived tumor cells, thereby causing epithelial cells to separate from each other, increasing the metastatic potential of tumor cells, and further resisting antitumor treatments [17, 18]. Accordingly, EMT plays a crucial role in the metastasis of epithelial tumors. Few studies have explored the prognostic value of EMT-related lncRNA in cancer patients [19]. The role of EMT-related lncRNA in ESCC and related mechanisms is little known.

This study comprehensively investigated and validated the prognostic value of EMT-related lncRNA in Chinese ESCC patients from the Gene Expression Omnibus (GEO) database by weighted gene coexpression network analysis (WGCNA), COX proportional hazard regression, and Kaplan-Meier survival analysis. Furthermore, risk stratification and a nomogram model were constructed to personalize and visualize the overall survival (OS) rates of ESCC patients. Additionally, the correlation between weighted EMT-related lncRNA with tumor microenvironment (TME) scores, immune checkpoints (ICs), and the Kyoto Encyclopedia of Genes and Genomes (KEGG) pathways was further explored.

## 2. Materials and Methods

### 2.1. ESCC Patients

**Figure 1:**
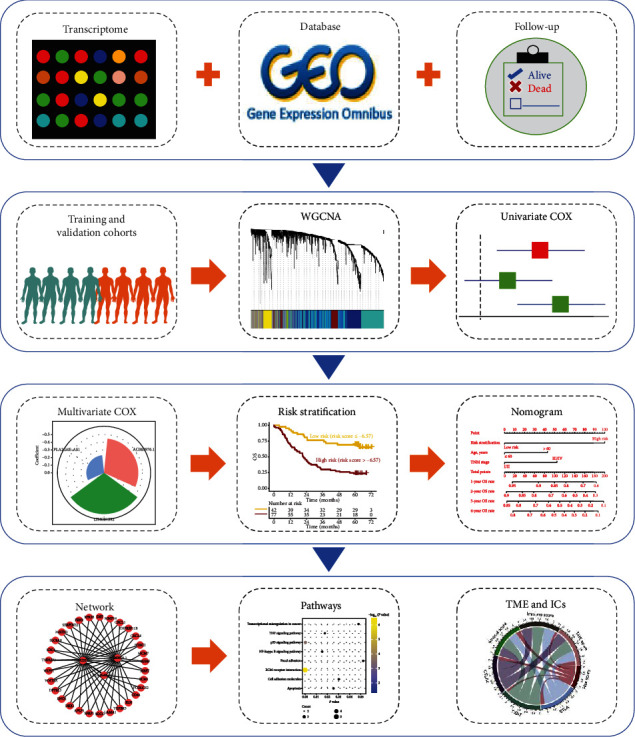
Schematic diagram of the study. The transcriptome sequencing data and clinical information of patients with esophageal squamous cell carcinoma (ESCC) were downloaded from the Gene Expression Omnibus (GEO) database. Different datasets were assigned as training and validation cohorts for weighted coexpression network analysis (WGCNA) and univariate COX proportional hazards regression analysis. Then, the epithelial-mesenchymal transition- (EMT-) related lncRNA determined by multivariate COX regression analysis was used for risk stratification and constructing a nomogram model. Finally, the pathways of EMT-related lncRNA and the correlation between lncRNA and tumor microenvironment (TME) or immune checkpoints (ICs) were explored.

The transcriptome data of 119 and 60 newly diagnosed ESCC patients in the GSE53624 and GSE53622 datasetswere downloaded from the GEO database (https://www.ncbi.nlm.nih.gov/geo/), assigned as training and validation cohorts, respectively. GSE53624 and GSE53622 datasets were all sampled from China. The clinical characteristics, including age, gender, tumor invasion depth (T), lymph node metastasis (N), tumor node metastasis (TNM) staging, tumor grade, overall survival (OS) time, and events, were obtained and listed in Table S1. The workflow of data analysis in this study was performed according to Figure 1. Since the GEO database is publicly available, no approval from the local ethics committee was required.

### 2.2. Acquisition of lncRNA and EMT-Related mRNA

A total of 17,936 lncRNA (version 35) were downloaded from the GENCODE database (https://www.gencodegenes.org/human/) [20]. Furthermore, the 50 hallmark gene sets and the “HALLMARK_EPITHELIAL_MESENCHYMAL_ TRANSITION” gene list, including 200 EMT-related mRNA, were obtained from the Gene Set Enrichment Analysis (GSEA) database (http://www.gsea-msigdb.org/gsea/msigdb/collections.jsp#H) [21, 22].

### 2.3. Weighted Gene Coexpression Network Analysis (WGCNA)

As an unsupervised machine learning, WGCNA was applied for investigating the correlation between genes. The *R* package “WGCNA” in *R* software (version 4.0.2, https://www.r-project.org/) was used to construct a weighted coexpression network between the lncRNA and EMT-related mRNA [23]. In the network, the pairwise Pearson coefficient was used to evaluate the coexpression weight among all genes. The power *β* of the soft threshold was used to confirm a scale-free network. Notably, the genes with similar expression patterns were clustered into the same color module in the unsupervised coexpression network.

### 2.4. Nomogram Model

The *R* packages “foreign” and “rms” in *R* software (version 4.0.2, https://www.r-project.org/) were used to construct a nomogram model to personalize and visualize the OS rate of ESCC patients [24, 25]. Each variable was assigned a point according to the nomogram model. Then, the total points were obtained by summing the points of all variables for determining the OS rate of an ESCC patient. Finally, time-dependent receiver operating characteristic (ROC) and calibration curves of the training and validation cohort were used to evaluate the accuracy of the nomogram model that predicted the OS rate.

### 2.5. Estimation of Tumor Microenvironment (TME) Score

The ESTIMATE algorithm was used to calculate the fraction of immune and stromal cells in ESCC tissues based on gene expression levels [26]. The ESTIMATE algorithm was performed using *R* package “estimate” to calculate TME, immune, and stromal scores in each ESCC patient.

### 2.6. Kyoto Encyclopedia of Genes and Genomes (KEGG) Pathways

“DOSE,” “http://org.Hs.eg.db,” “topGO,” and “clusterProfiler” packages in *R* software (version 4.0.2, https://www.r-project.org/) were used to obtain the KEGG pathways of EMT-related lncRNA in ESCC patients.

### 2.7. Statistical Analysis

All statistical analysis was performed using *R* software (version 4.0.2, https://www.r-project.org/). A chi-square and Fisher tests were used to compare differences between two groups of categorical variables, as appropriate. Univariate and multivariate Cox proportional hazard regression analysis was performed using package “survival.” The “surv_cutpoint” function in the *R* package “survminer” was used to determine the optimal cut-point of a gene (Figure S1). Kaplan-Meier curves were compared by the log-rank test. The *R* package “survRM2” was used to obtain the restricted mean survival time (RMST). Correlation coefficients between two quantitative variables were obtained by Pearson's method. The “survivalROC” package determined the area under the curve (AUC) in the time-dependent ROC curve. A two-tailed *P* value <0.05 and a *P* value <0.1 were considered statistically significant and a clear trend, respectively.

## 3. Results

### 3.1. WGCNA for lncRNA and EMT-Related mRNA in ESCC Patients

**Figure 2:**
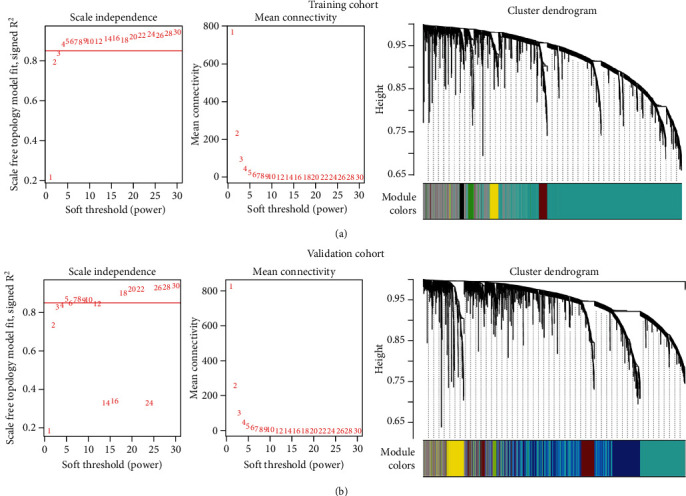
Construction of WGCNA between lncRNA and EMT-related genes in the training (a) and validation (b) cohorts. The soft threshold was obtained when the scale-free topology model was fitted to 0.85 (a). The dendrogram was constructed by hierarchical clustering of coexpressed genes (b). The different colors below the dendrogram represented the modules corresponding to the coexpression of lncRNA and EMT-related genes.

As shown in Table S1, the clinical characteristics were balanced between GSE53624 and GSE53622 datasets (*P* > 0.05). To identify EMT-related lncRNA in ESCC patients, 188 EMT-related mRNA and 5,506 lncRNA were included in the construction of WGCNA, and the workflow for data analysis was shown in Figure 1. The soft thresholds for building a scale-free network of training and validation cohorts were set to 4 and 5, respectively (Figures 2(a) and 2(b)). Then, a total of 3,900 lncRNA were coexpressed with EMT-related mRNA in the training cohort, which was distributed in 6 modules, including black, blue, brown, green, grey, and turquoise modules (Figure 2(a)). Moreover, 3,742 lncRNA and EMT-related mRNA were showed in 11 coexpression modules, including black, blue, green, greenyellow, grey, magenta, pink, purple, red, turquoise, and yellow, in the validation cohort (Figure 2(b)). Therefore, 3,900 and 3,742 EMT-related lncRNA in the training and validation cohorts, respectively, were used for the following univariate and multivariate COX regression analysis. Notably, the percent of overlapping EMT-related lncRNA between training and validation cohorts was 78.1% (3,045/3,900) and 81.4% (3,045/3,742), respectively.

### 3.2. Univariate and Multivariate COX Regression Analysis

**Figure 3:**
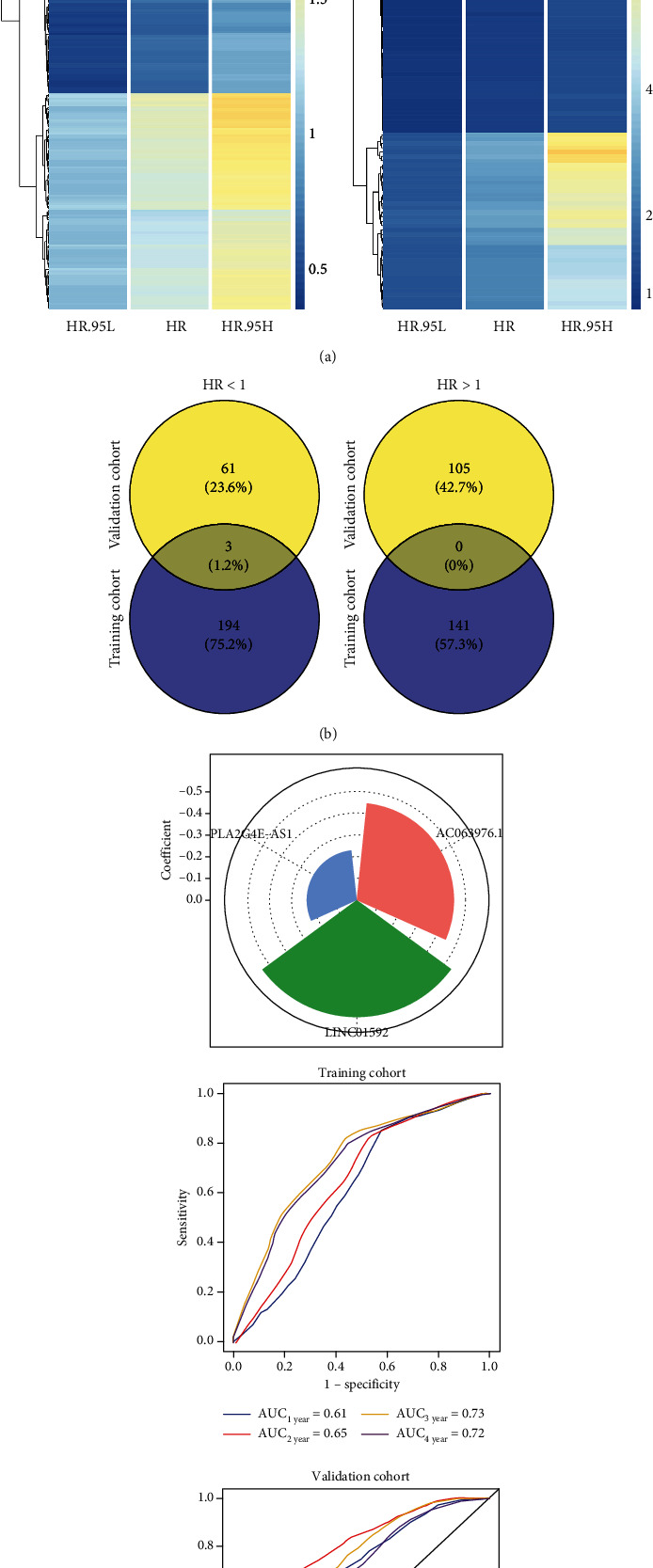
Univariate and multivariate COX regression analysis of EMT-related lncRNA in the training and validation cohorts. (a) The heatmaps displayed the hazard ratio (HR) and 95% confidence interval (CI) of EMT-related lncRNA with *P* < 0.05 by univariate COX regression analysis in the training (a) and validation (c) cohorts. Blue to red in the color scale represented HR from low to high. HR.95 L: the low value in the 95% confidence interval of HR; HR.95H: the high value in the 95% confidence interval of HR. (b) The overlapping number of EMT-related lncRNA with HR < 1 (a) or HR > 1 (c) in the training and validation cohorts. (c) Multivariate COX regression analysis of EMT-related lncRNA. The contribution of EMT-related lncRNA to the overall survival (OS) of ESCC patients in the training cohort (upper panel). The time-dependent receiver operator characteristic (ROC) curves of the training (b) and validation (d) cohorts were used to evaluate the performance of the multivariate COX regression model.

After univariate COX regression analysis, 338 and 169 EMT-related lncRNA were significantly associated with the OS of ESCC patients in the training and validation cohorts, respectively (*P* < 0.05, Figure 3(a)). To further confirm that EMT-related lncRNA had prognostic value in both cohorts, lncRNA with a hazard ratio (HR) > 1 or HR<1 in the univariate regression model was overlapped between the training and validation cohorts, and the results showed that 3 EMT-related lncRNA expression, including PLA2G4E-AS1, AC063976.1, and LINC01592, significantly correlated with the favorable OS of ESCC patients (HR < 1, Figure 3(b)). Then, multivariate COX regression analysis was used for weighted combination of AC063976.1, LINC01592, and PLA2G4E-AS1, which indicated that LINC01592 contributed the greatest to the OS of ESCC patients (coefficient = −0.54). Notably, the time-dependent ROC curve results demonstrated that the multivariate COX regression model performs well in the training cohort (AUC > 0.60, Figure 3(c)). This finding was confirmed in the validation cohort (AUC ≥ 0.70, Figure 3(c)).

### 3.3. Establishment of Risk Stratification for ESCC Patients

**Figure 4:**
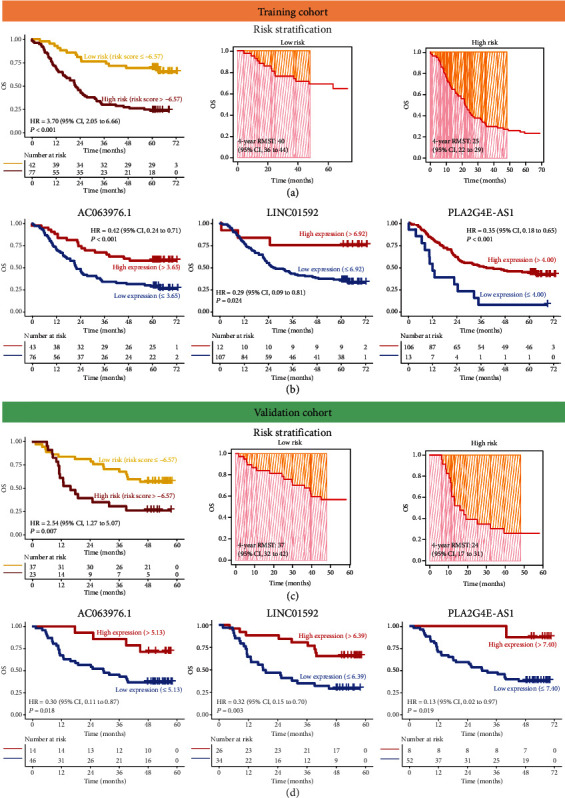
The weighted combination of AC063976.1, LINC01592, and PLA2G4E-AS1 was used for risk stratification of ESCC patients. (a, c) Kaplan-Meier curves (a) and 4-year restricted mean survival time (RMST) (b)–(d) for low- and high-risk groups of ESCC patients in the training (a) and validation (c) cohorts. (b, d) Kaplan-Meier curves were plotted based on the expression levels of AC063976.1 (a), LINC01592 (b), and PLA2G4E-AS1 (c, d) in training (b) and validation (d) cohorts.

**Figure 5:**
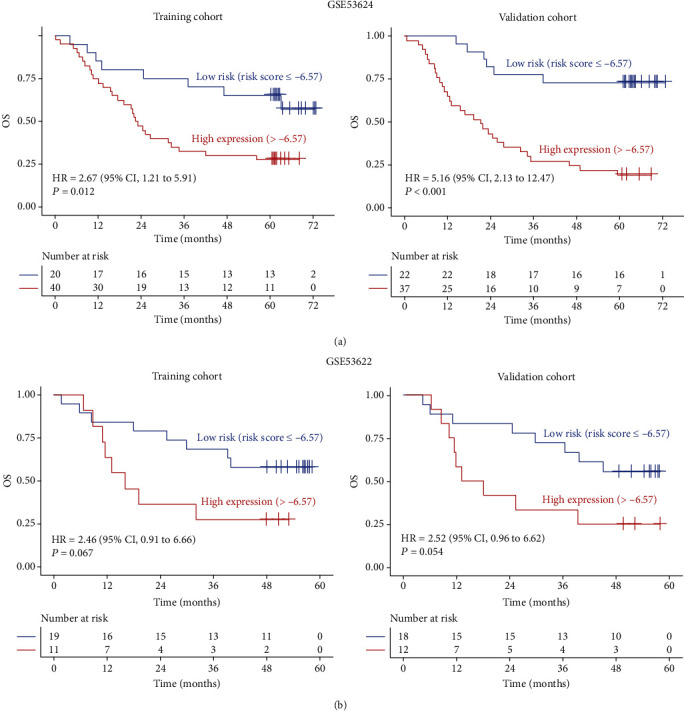
The risk stratification was performed in GSE53624 (a) and GSE53622 (b) datasets based on the combination of AC063976.1, LINC01592, and PLA2G4E-AS1. Microsoft Excel 2016 was used to randomly select a portion of samples in each dataset as training cohort (a) and then treat the other samples as validation cohort (b).

**Figure 6:**
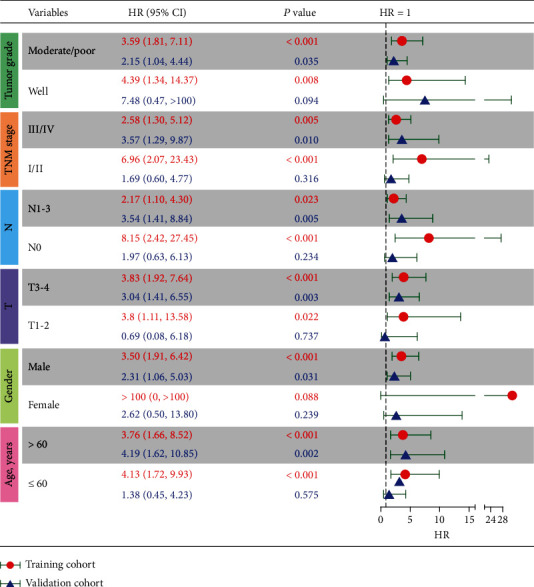
Subgroup analysis of risk stratification in different tumor grades, the tumor, node, metastasis (TNM) stage, lymph node metastasis (N), tumor invasion depth (T), gender, and age in the training and validation cohorts. CI: confident interval; HR: hazard ratio.

To establish a risk stratification for ESCC patients, we first obtain the risk score based on the coefficients of multivariate COX regression, and the formula for calculating the risk score was as follows: risk score = −0.45 × (expression level of AC063976.1) − 0.54 × (expression level of LINC01592) − 0.23 × (expression level of PLA2G4E − AS1) (Figure 3(c)). Based on the optimal prognostic cut-point of risk score -6.57, ESCC patients were divided into high- and low-risk groups. ESCC patients with high risk were significantly associated with poor OS in the training cohort (HR = 3.70, 95% confidence interval (CI): 2.05 to 6.66, *P* < 0.001, Figure 4(a)). This result was confirmed in the validation cohort (HR = 2.54, 95% CI: 1.27 to 5.07, *P* = 0.007, Figure 4(c)). Furthermore, high-risk ESCC patients had a shorter RMST than low-risk patients in the training cohort (4-year RMST: 25 (95% CI: 22 to 29) vs. 40 (95% CI: 36 to 44) months) (Figure 4(a)). This result was again confirmed in the validation cohort (4-year RMST: 24 (95% CI: 17 to 31) vs. 37 (95% CI: 32 to 42) months) (Figure 4(c)). Interestingly, The Kaplan-Meier curves indicated that high expression of AC063976.1, LINC01592, and PLA2G4E-AS1 correlated with the favorable OS of ESCC patients in both the training and validation cohorts (*P* < 0.05, Figures 4(b) and 4(d)). Importantly, risk stratification was an independent prognostic predictor for ESCC patients by univariate and multivariate COX regression analysis in the training cohort (HR = 3.89, 95% CI: 2.13 to 7.11, *P* < 0.001, Table 1). This was again confirmed in the validation cohort (HR = 2.73, 95% CI: 1.29 to 5.78, *P* = 0.008, Table 1). In order to determine whether risk stratification has prognostic significance in a random population, Microsoft Excel 2016 was further used to randomly select a portion of samples in each dataset as a training cohort and then treat the other samples as a validation cohort. Patients with a high-risk score were associated with a poor OS in the training cohort of the GSE53624 dataset (HR = 2.67, 95% CI: 1.21 to 5.91, *P* = 0.012). This result was confirmed in the validation cohort of GSE53624 dataset (HR = 5.16, 95% CI: 2.13 to 12.47, *P* < 0.001) (Figure 5(a)). Interestingly, high-risk patients had a shorter OS than low-risk patients in the training cohort of the GSE53622 dataset; although, it is not statistically significant at that point (HR = 2.46, 95% CI: 0.91 to 6.66, *P* = 0.067). This finding was again confirmed in the validation cohort of GSE53622 dataset (HR = 2.52, 95% CI: 0.96 to 6.62, *P* = 0.054) (Figure 5(b)). This might be due to the small sample size in the GSE53622 dataset. To confirm the risk stratification that could better predict prognosis in which part of the population of ESCC patients, we conducted a subgroup analysis. There is a clear trend in the training and validation cohorts that risk stratification can better predict the prognosis in patients with TNM III/IV stage, N1-3, T3-4, male, and >60 years old. The prognosis can be predicted well through risk stratification regardless of the tumor grade (Figure 6).

### 3.4. Construction of a Nomogram Visualizing and Personalizing the OS Rate of ESCC Patients

**Figure 7:**
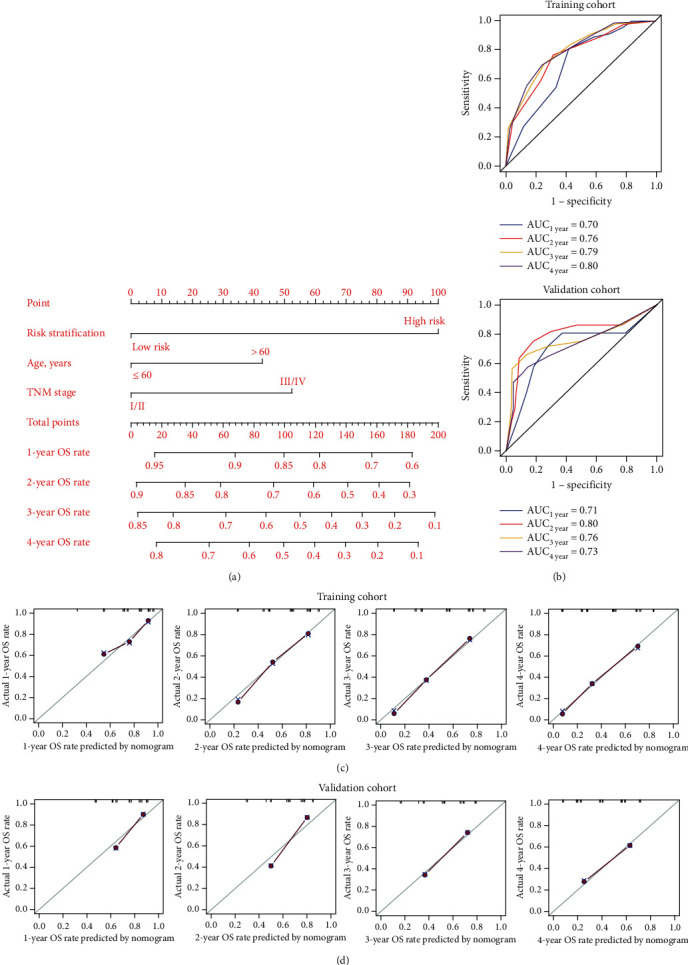
Construction of the nomogram model. (a) The combination of risk stratification, age, and TNM stage visualized and personalized the OS rate of ESCC patients. After a point was assigned to risk stratification, age, and TNM stage of each patient according to the nomogram model, the total points were obtained to predict the OS rate of ESCC patients. (b) The time-dependent receive operating characteristic curve (ROC) curve was used to evaluate the performance of the nomogram model in the training (a, b) and validation (c, d) cohorts. (c d) The calibration curves were used to evaluate the performance of the nomogram model in predicting the OS rate in training (c) and validation (d) cohorts. The criterion for good performance of the nomogram model was that the predicted OS rate was relatively consistent with the actual OS rate. AUC: the area under the curve.

Univariate and multivariate COX regression analysis was used to identify independent prognosis factors for constructing a nomogram model. In addition to risk stratification, age and TNM stage were independent prognostic factors for ESCC patients in both the training and validation cohorts (HR > 1, *P* < 0.1, Table 1). Accordingly, a nomogram model constructed by the risk stratification, age, and TNM stage could visualize and personalize the 1-, 2-, 3-, and 4-year OS rates of ESCC patients (Figure 7(a)). Details of the points for the variables and OS rates in the nomogram model were listed in Table S2. The time-dependent ROC and calibration curves were further used to evaluate the predicted performance of the nomogram model. Notably, the time-dependent ROC curves illustrated that all the AUCs were ≥0.70 in both the training and validation cohorts (Figure 7(b)). Moreover, calibration curves indicated that the 1-, 2-, 3-, and 4-year OS rates predicted in the nomogram model were highly in line with actual observations in both the training and validation cohorts (Figures 7(c) and 7(d)). These results suggested that the nomogram model had good performance in predicting the OS rate of ESCC patients.

### 3.5. KEGG Pathways for EMT-Related lncRNA

**Figure 8:**
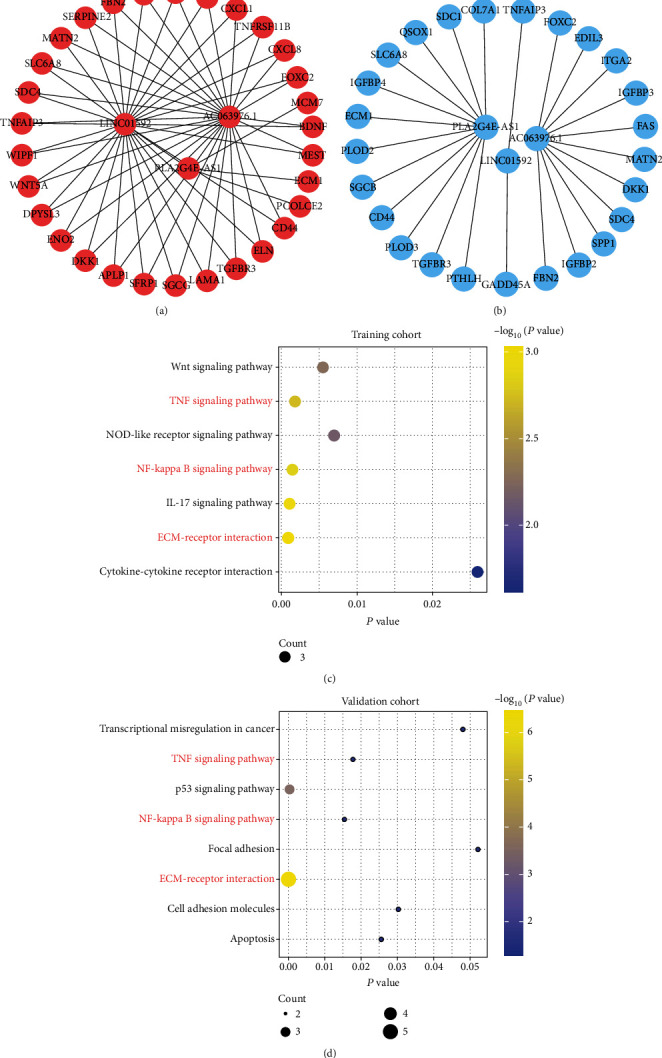
Kyoto Encyclopedia of Genes and Genomes (KEGG) pathways for the EMT-related lncRNA. (a, b) mRNA that coexpressed with EMT-related lncRNA was obtained from WGCNA in the training (a) and validation (b) cohorts. (c, d) The KEGG pathways for the EMT-related lncRNA in training (c) and validation (d) cohorts. The pathways marked in red represented the overlapped pathways enriched by EMT-related lncRNA in both the training and validation cohorts.

Based on WGCNA, 57 and 25 EMT-related genes were coexpressed with AC063976.1, LINC01592, or PLA2G4E-AS1 in the training and validation cohorts, respectively (Figure 8(a)). Then, the KEGG was applied to identify the significant pathway associated with the AC063976.1, LINC01592, or PLA2G4E-AS1 in ESCC patients. The results showed that in the training and validation cohorts, a total of 7 and 8 pathways were enriched, respectively. And three overlapped pathways, including TNF signaling pathway, NF-kappa B signaling pathway, and ECM-receptor interaction, were enriched in both cohorts (Figures 8(c) and 8(d)).

### 3.6. The Risk Score Was Positively Correlated with the TME Score and Immune Checkpoints (ICs)

**Figure 9:**
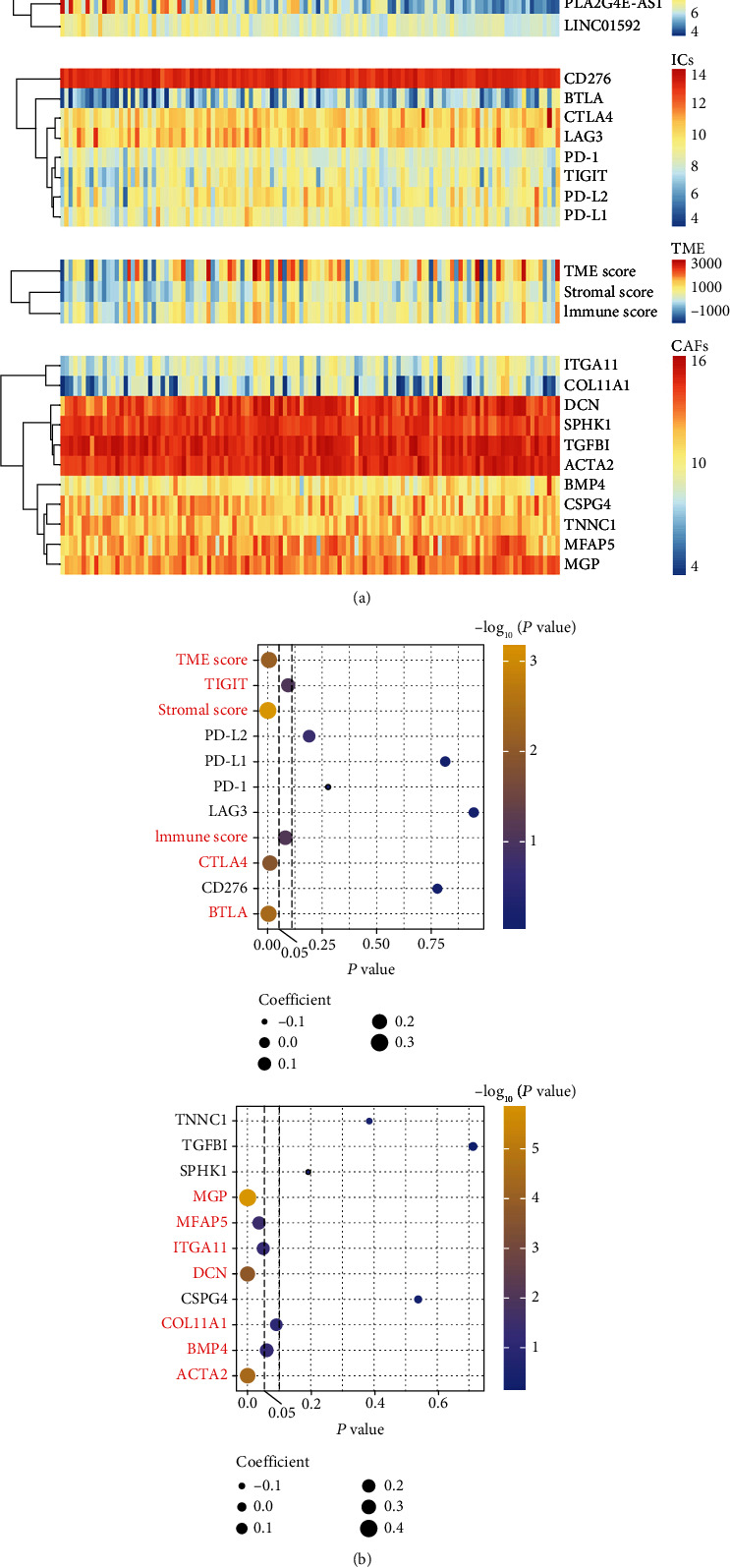
The correlation between risk score estimated by nomogram and tumor microenvironment (TME) and immune checkpoints (ICs) in the training cohort. (a) Distribution of risk score, TME score, ICs, and cancer-associated fibroblast (CAF) related gene expression level. *R* package “estimate” was used to calculate TME, immune, and stromal scores in each ESCC patient based on gene expression levels. (b) The relationship between risk score and TME, ICs (a) and CAFs (b). The size of the dot represents the size of the correlation coefficient, and the color scale from blue to yellow represents the *P* value from large to small.

Ample reports found that EMT-related genes were enriched in TME, whereas little was known about these genes in ESCC. This prompted us to investigate further the relationship between the risk score based on EMT-related lncRNA and TME score and the expression levels of ICs. TME score was composed of stromal score and immune score. The results suggested that risk score was positively correlated with TME score (*R* = 0.25, *P* = 0.007). Further analysis found that the risk score had a positive correlation with immune score (*R* = 0.16, *P* = 0.081); although, statistical significance was not reached at this point. Because ICs is closely related to immune score, we further analyzed the correlation between risk score and ICs. Notably, risk score had a significantly positive correlation with BTLA (*R* = 0.26, *P* = 0.004) and CTLA4 (*R* = 0.23, *P* = 0.012). What is more, there was a clear trend suggested that the risk score was positively correlated with TIGIT (*R* = 0.15, *P* = 0.093); although, statistical significance was not reached at this point. However, there was no significant correlation between risk score and PD-1, PD-L1, PD-L2, LAG3, and CD276 (*P* > 0.1). Interestingly, risk score was also positively correlated with stromal scores (*R* = 0.31, *P* < 0.001) (Figures 9(a) and 9(b)). We further analyzed the correlation between risk score and cancer-associated fibroblast- (CAFs-) related genes. The results suggested that the risk score had a significant positive correlation with MGP (*R* = 0.43, *P* < 0.001), MFAP5 (*R* = 0.19, *P* = 0.036), ITGA11 (*R* = 0.18, *P* = 0.050), DCN (*R* = 0.34, *P* < 0.001), or ACTA2 (*R* = 0.36, *P* < 0.001). Moreover, there was a clear trend showing that risk score was positively correlated with COL11A1 (*R* = 0.16, *P* = 0.091) and BMP4 (*R* = 0.17, *P* = 0.060); although. the data were not yet significant enough at this point. However, there is no significant correlation between risk score and SPHK1, CSPG4, TGFBI, and TNNC1 (*P* > 0.1) (Figures 9(a) and 9(b)).

## 4. Discussion

ESCC is the prevalent histological subtype of esophageal cancer, with a poor prognosis and prone to distant metastasis [3, 27]. Therefore, exploring potential biomarkers is essential for managing and predicting the prognosis of ESCC patients. Increasing evidence suggests that EMT is highly correlated with cancer progression and metastasis [28]. In recent years, a few studies have investigated the role of EMT-related lncRNA in the prognosis and progression of ESCC [29, 30]. However, based on next-generation transcriptome sequencing, a comprehensive assessment of the prognostic importance, risk stratification, and visualization of OS rates by EMT-related lncRNA in ESCC was little known.

In this study, based on the analysis of two large datasets in the GEO database, the results suggested that the high expression of AC063976.1, LINC01592, or PLA2G4E-AS1 was significantly associated with favorable OS in ESCC patients. In addition, KEGG results indicated that AC063976.1, LINC01592, or PLA2G4E-AS1 were mainly enriched in the TNF signaling pathway, NF-kappa B signaling pathway, and ECM-receptor interaction. AC063976.1, as a novel EMT-related lncRNA, has not yet been explored in cancer. For the first time, we revealed that upregulation of AC063976.1 corrected with a favorable OS of ESCC patients. Li et al. reported that LINC01592 was a protective factor for ESCC patients [31], which was consistent with this study. Furthermore, LINC01592 contributed the greatest to the OS of ESCC patients. Although PLA2G4E-AS1 was downregulated in thyroid carcinoma, its prognostic importance in cancer patients has not been elucidated [32]. The results of this study demonstrated that the high expression of PLA2G4E-AS1 could predict favorable OS of ESCC patients. These findings will provide prognostic information for further exploring the functions and mechanisms of AC063976.1, LINC01592, or PLA2G4E-AS1 in ESCC in the future.

Risk stratification plays a vital role in guiding clinical treatment and management of cancer patients [33]. Notably, the risk stratification constructed by a weighted combination of AC063976.1, LINC01592, and PLA2G4E-AS1 divided ESCC patients into low- and high-risk groups, implying it was also an independent prognostic predictor for ESCC patients. Interestingly, a subgroup analysis found that risk stratification was mainly performed in ESCC patients with TNM III/IV stage, N1-3, T3-4, male, or >60 y. Furthermore, the risk stratification could be used regardless of the tumor grade. Notably, a nomogram model established by the risk stratification, age, and TNM stage could display and visualize the 1-, 2-, 3-, and 4-year OS rates of ESCC patients, which might contribute to the management of individualized treatment.

Previous studies reported that TME was associated with EMT in cancers [34]. Moreover, the stromal microenvironment and CAFs play an important role in EMT [35–38]. Hence, the relationship between risk scores calculated by AC063976.1, LINC01592, and PLA2G4E-AS1 and TME was further investigated. In this study, the risk score was positively correlated with TME, immune, and stromal scores. Furthermore, the risk score had a significant positive correlation with CAF genes, including MGP, ITGA11, DCN, ACTA2, COL11A1, or BMP4. However, high expression levels of ICs usually lead to T cell exhaustion in cancers [39, 40]. Interestingly, there was also a positive correlation between risk score and ICs, including BTLA, TIGIT, and CTLA4. These results can be interpreted that the antitumor effect of the high level of immune cell infiltration is offset by the strong immunosuppressive pathway activated by upregulated IC proteins [34, 41] and might provide the likelihood of immunotherapy for high-risk ESCC patients.

This study had several limitations: first, the results of the analysis and validation of transcriptome sequencing data in this study were based on publicly available datasets. Therefore, some important clinical information was incomplete, such as treatment options, which might produce potential biases in conclusions. Secondly, this study did not provide additional validation by original ESCC samples from our clinical center. Finally, EMT-related lncRNA should be validated through in vivo and in vitro experiments in the future.

## 5. Conclusions

We demonstrated that based on risk stratification constructed by PLA2G4E-AS1, AC063976.1, and LINC01592, ESCC patients were divided into low- and high-risk groups. Moreover, a nomogram model established by the risk stratification, age, and TNM stage could display and visualize the 1-, 2-, 3-, and 4-year OS rates of Chinese ESCC patients. In addition, the risk score was positively correlated with the TME score, ICs, and CAFs. These findings might provide deep insight for personalized prognosis prediction by EMT-related lncRNA in Chinese ESCC patients, and the three lncRNAs might be potential biomarkers for designing novel therapy.

## Figures and Tables

**Table 1:** Uni- and multivariate regression analysis in ESCC patients.

Variables	Univariate COX regression				Multivariate COX regression			
	Training cohort		Validation cohort		Training cohort		Validation cohort	
	HR (95% CI)	*P* value	HR (95% CI)	*P* value	HR (95% CI)	*P* value	HR (95% CI)	*P* value
Risk stratification								
Low risk	Reference		Reference		Reference		Reference	
High risk	3.70 (2.05, 6.66)	< 0.001	2.54 (1.27, 5.07)	0.009	3.89 (2.13, 7.11)	< 0.001	2.73 (1.29, 5.78)	0.008
Age, years								
≤ 60	Reference		Reference		Reference		Reference	
>60	1.58 (1.00, 2.50)	0.051	1.97 (0.98, 3.97)	0.057	1.77 (1.10, 2.85)	0.018	1.95 (0.95, 4.00)	0.067
Gender								
Female	Reference		Reference					
Male	0.83 (0.47, 1.46)	0.513	0.71 (0.31, 1.64)	0.424				
T								
T1-2	Reference		Reference					
T3-4	0.96 (0.57, 1.64)	0.891	1.55 (0.60, 4.01)	0.369				
N								
N0	Reference		Reference		Reference		Reference	
N1-3	2.16 (1.32, 3.54)	0.002	2.02 (0.99, 4.12)	0.053	1.11 (0.57, 2.17)	0.758	2.47 (0.66, 9.24)	0.179
TNM stage								
I/II	Reference		Reference		Reference		Reference	
III/IV	2.16 (1.32, 3.54)	0.002	2.01 (1.01, 4.01)	0.046	1.91 (0.99, 3.71)	0.055	0.80 (0.22, 2.99)	0.742
Tumor grade								
Well	Reference		Reference					
Moderate/poor	1.00 (0.56, 1.80)	0.989	2.04 (0.62, 6.70)	0.239				

CI: confidence interval; HR: hazard ratio; N: lymph node metastasis; T: tumor invasion depth.

## Data Availability

The original contributions presented in the study are included in the article/Supplementary Material, and further inquiries can be directed to the corresponding authors.

## Abbreviations

AUC: Area under the curve
CI: Confidence interval
EMT: Epithelial-mesenchymal transition
ESCC: Esophageal squamous cell carcinoma
GEO: Gene expression omnibus
GSEA: Gene set enrichment analysis
HR: Hazard ratio
IC: Immune checkpoint
KEGG: Kyoto Encyclopedia of Genes and Genomes
lncRNA: Long noncoding RNA
OS: Overall survival
RMST: Restricted mean survival time
ROC: Receiver operating characteristic
TME: Tumor microenvironment
TNM: Tumor invasion depth, lymph node metastasis, tumor node metastasis
WGCNA: Weighted coexpression network analysis.

## Supplementary Materials

Supplementary Materials Figure S1: the optimal cut-points for risk score, AC063976.1, LINC01592, and PLA2G4E-AS1 in the training (a) and validation (b) cohorts. Table S1: clinical characteristics of ESCC patients. Table S2: the points for the nomogram model.
