# Supplementary material for: Identification of Epithelial-Mesenchymal Transition- (EMT-) Related LncRNA for Prognostic Prediction and Risk Stratification in Esophageal Squamous Cell Carcinoma
Source: Dis Markers. 2021 Oct 19;2021:5340240. doi: 10.1155/2021/5340240 (PMC8548124; doi:10.1155/2021/5340240)
Supplement: Supplementary Materials — Figure S1: the optimal cut-points for risk score, AC063976.1, LINC01592, and PLA2G4E-AS1 in the training (a) and validation (b) cohorts. Table S1: clinical characteristics of ESCC patients. Table S2: the points for the nomogram model. [file 5340240.f1.pdf]

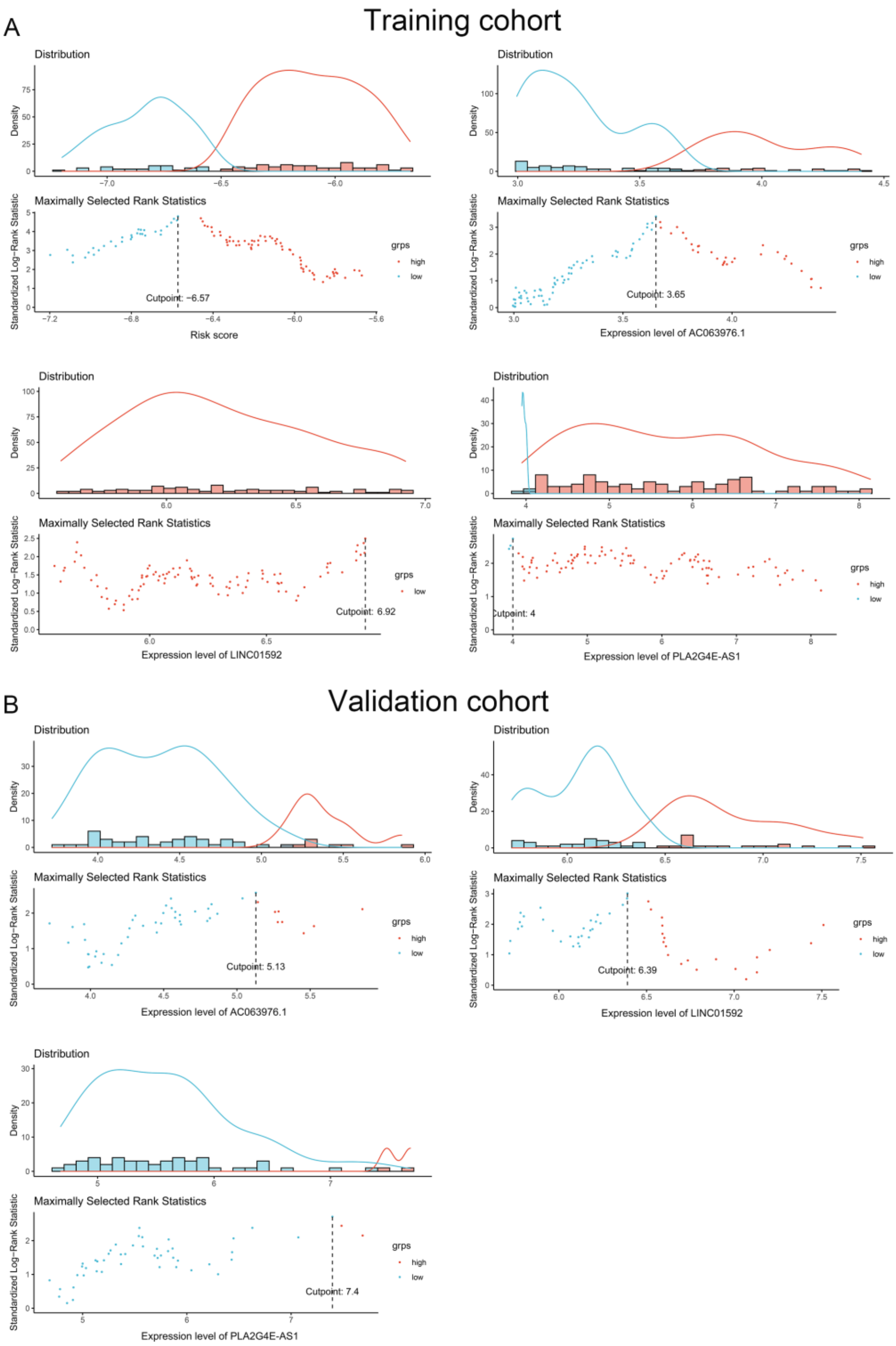

Fig. S1. The optimal cut-points for risk score, AC063976.1, LINC01592, and PLA2G4E-AS1 in the training (A) and validation (B) cohorts.

Table S1. Clinical characteristics of ESCC patients.

**Variables**	**Training cohort, n (%)**	**Validation cohort, n (%)**	***P*-value**
Number	119	60	NA
Age, years			0.393
≤ 60	69 (58.0)	30 (50.0)	
> 60	50 (42.0)	30 (50.0)	
Gender			0.858
Female	21 (17.6)	12 (20.0)	
Male	88 (82.4)	48 (80.0)	
T			0.546
T1-2	28 (23.5)	11 (18.3)	
T3-4	91 (76.5)	49 (81.7)	
N			0.829
N0	54 (45.4)	29 (48.3)	
N1-3	65 (54.6)	31 (51.7)	
TNM stage			0.169
I/II	53 (44.5)	34 (56.7)	
III/IV	66 (55.5)	26 (43.3)	
Tumor grade			0.612
Well	23 (19.3)	9 (15.0)	
Moderate/poor	96 (80.7)	51 (85.0)	

NA, not available; N, lymph node metastasis; T, tumor invasion depth; TNM stage, the Tumor, Node, Metastasis stage.

Table S2. The points for nomogram model.

Variables	Points	OS time	OS rate	Total points
Risk stratification		1 year		
Low risk	0		0.95	15
High risk	100		0.90	68
Age, years			0.85	99
≤ 60	0		0.80	123
> 60	43		0.70	157
TNM stage			0.60	183
I/II	0	2 year		
III/IV	52		0.90	4
			0.85	35
			0.80	58
			0.70	93
			0.60	119
			0.50	141
			0.40	161
			0.30	181
		3 year		
			0.85	4
			0.80	27
			0.70	62
			0.60	88
			0.50	110
			0.40	131
			0.30	150
			0.20	172
			0.10	198
		4 year		
			0.80	17
			0.70	51
			0.60	77
			0.50	99

0.40	120
0.30	140
0.20	161
0.10	187
